# Incorporation of Ruxolitinib in the Management of Refractory/Relapsed Hodgkin Lymphoma: Where Do We Stand?

**DOI:** 10.46989/001c.87501

**Published:** 2023-08-28

**Authors:** Ahmed H. Al Sharie, Balqis M. Abu Mousa, Ahmad O. Alomari

**Affiliations:** 1 Faculty of Medicine, Jordan University of Science & Technology, Irbid 22110, Jordan

**Keywords:** Hodgkin lymphoma, Refractory, Relapsed, Ruxolitinib

Hodgkin lymphoma (HL) is a highly sensitive neoplasm to systemic combined chemotherapy. Despite the optimal response rate seen in HL patients, 10-15% exhibit a refractory/relapsed (R/R) disease course with a dismal prognosis. After treatment failure, high-dose chemotherapy and with autologous stem cell transplantation are warranted. Even in the presence of newly tailored agents, many R/R HL cases are regarded as palliative.^1^ Recent advances in transcriptome profiling have elucidated many altered pathways in HL, including immune evasion, NF-κB, PI3K/AKT/mTOR, and JAK/STAT. The latter is observed due to the classical 9p and 2p gains in HL.^2^ JAK is a cytoplasmic tyrosine kinase that modulates gene transcription by phosphorylating STAT factors. Although alterations in JAK/STAT pathway are commonly detected in myeloproliferative disorders and myeloid neoplasms, they play an important role in the tumorigenesis of HL. In the past decade, the therapeutic potential of JAK/STAT inhibition in HL has been conceptualized and popularized in many preclinical studies. Such rationale resulted in a group of clinical trials to evaluate the proposed hypothesis. Since ruxolitinib, an oral, selective inhibitor of JAK1/JAK2, was approved by the FDA in 2011 for myeloproliferative disorders, it was used in HL trials.

In 2014, Kim et al. published the first preliminary data of a multi-center, single-agent pilot study (NCT01965119) evaluating the 28-day cycle of 20 mg twice daily of ruxolitinib in patients with R/R HL. The study, in which 13 HL patients were enrolled, was finalized and published in 2019.^3^ The response rate was 54%, with one complete remission, one stable disease, and five partial remissions, with a median response duration of 5.6 months. Ruxolitinib showed an acceptable safety profile, with manageable grade 1 and 2 adverse events. Another multi-center, open-label, phase II clinical trial (NCT01877005) conducted at the Lymphoma Study Association (LYSA) centers evaluated ruxolitinib in 33 patients with advanced R/R HL.^4^ The response rate was poor in those who received more than 6 cycles, with only three patients exhibiting partial response and 11 with stable disease. The median response duration, 7.7 months, was longer than the previous study. The reported median progression-free and median overall survival were 3.5 and 27.1 months, respectively. Ruxolitinib showed limited toxicity and an acceptable safety profile, in which 42.4% of the participants had a side event, with only one that led to treatment discontinuation. The JeRiCHO trial (NCT02164500), a 2-stage phase 2 design, open-label, multi-center clinical trial, evaluated the effect of 25 mg twice daily ruxolitinib in R/R classical HL.^5^ The trial enrolled 12 patients in the first stage. Only one had a partial response, with the rest having stable (three patients) or progressive (six patients) disease course. Progression-free survival was 3.6 months, while the 1-year overall survival was 50.6%, with a favorable toxicity profile. The reported data suggest that ruxolitinib has a modest short-lived, and limited potential in HL patients, with a highly acceptable safety profile. Additional studies with expanded cohorts are warranted to conclude.

The observed effect could be augmented if ruxolitinib got incorporated with other agents. Hence, the synergetic potential of combining ruxolitinib with other medications in R/R HL is of great interest for clinical evaluation. In Phase I/II, multi-center, open-label trial (NCT03681561) evaluating the efficacy, safety, and tolerability of ruxolitinib combined with nivolumab for R/R HL after immune checkpoint inhibitor therapy failure,^6^ a total of 19 patients were enrolled, of which 16 were eligible for response evaluation. The best overall response rate was 75%. Three patients experienced complete remission, two a partial remission, and six patients remained in stable disease. The 1-year progression-free survival was 64%. The combination therapy showed proper safety and tolerability; the most adverse events were grade 1 and 2, with a minority requiring hospitalization or having immune-related events. The reported data are preliminary but show a promising potential for dual JAK2 and PD-1 blockage, with encouraging synergistic activity. In an ongoing trial evaluating ruxolitinib with etoposide and corticosteroids in heavily pretreated HL and non-HL patients, the preliminary report showed only 1 enrolled patient with HL exhibiting a stable disease course and progression-free survival of 102 days without adverse events.^7^ The combinations of ruxolitinib with pembrolizumab (NCT04016116) or bortezomib (NCT02613598) in HL are being investigated in two distinct ongoing clinical trials.

In conclusion, ruxolitinib monotherapy in R/R HL shows limited efficacy, safe profile, and little toxicity. Table 1 summarizes all the published data on the utilization of ruxolitinib in R/R HL. In contrast, combination therapy with immune check-point inhibitors or other chemotherapeutic agents could exhibit significant efficacy; it is too early to conclude such fact without comprehensive trials with large cohorts and detailed molecular studies.

**Table 1.** Summary of clinical trials evaluating the safety and efficacy of ruxolitinib or ruxolitinib-based regimen in R/R HL.

**Regimen type**	**Number of patients enrolled**	**Regimen dosages**	**Patients’ response**	**Median response duration (months)**	**Adverse events grading**	**NCT number**	**Reference**
**Ruxolitinib monotherapy**	13	Ruxolitinib: 20 mg twice daily for a 28-day cycle.	6 (one CR, five PR, and one SD)	5.6	G1-G2	NCT01965119	^3^
	33	Ruxolitinib: 20 mg twice daily for a 28-day cycle.	20 (six CR, three PR, and 11 SD*)	7.7	G1-G3	NCT01877005	^4^
	12	Ruxolitinib: 25 mg twice daily for a 28-day cycle.	5 (Zero CR, two PR, and three SD)	5.6	G1-G4	NCT02164500	^5^
**Ruxolitinib-based combined regimens**	16	Ruxolitinib: 10, 15, and 30 mg twice daily and nivolumab:3mg/kg IV every 2 weeks) for 2 years.	12 (three CR, two PR, and six SD)	12.5	G1-G3	NCT03681561	^6^
	1	Ruxolitinib: 10 or 5 mg twice daily for a 28-day cycle and etoposide: 50mg/m2 with prednisone: 0 or 5 mg/kg daily.	1 (one SD)	-	No adverse events	Not registered.	^7^

CR: complete response, PR: partial remission, and SD: stable disease.

*SD was transient.

## Ethical Approval

Not applicable.

## Consent to participate/Informed Consent

Not applicable.

## Consent for publication

Not applicable.

## Competing interests/ Conflict of Interest

Not applicable.

## Authors’ Contribution – CRediT taxonomy

*Conceptualization*: Ahmed H. Al Sharie, Balqis M. Abu Mousa, Ahmad O. Alomari; *Methodology*: Ahmed H. Al Sharie, Balqis M. Abu Mousa, Ahmad O. Alomari; *Formal analysis and investigation*: N/A; *Writing - original draft preparation*: Ahmed H. Al Sharie, Balqis M. Abu Mousa, Ahmad O. Alomari; *Writing - review and editing*: Ahmed H. Al Sharie; *Funding acquisition*: N/A; *Resources*: N/A; *Supervision*: Ahmed H. Al Sharie.
